# Genotype–phenotype correlation study in 364 osteogenesis imperfecta Italian patients

**DOI:** 10.1038/s41431-019-0373-x

**Published:** 2019-03-18

**Authors:** Margherita Maioli, Maria Gnoli, Manila Boarini, Morena Tremosini, Anna Zambrano, Elena Pedrini, Marina Mordenti, Serena Corsini, Patrizia D’Eufemia, Paolo Versacci, Mauro Celli, Luca Sangiorgi

**Affiliations:** 1 Department of Medical Genetics and Rare Orthopaedic Diseases, IRCCS Istituto Ortopedico Rizzoli, Bologna, Italy; 2 CLIBI Laboratory, IRCCS Istituto Ortopedico Rizzoli, Bologna, Italy; 3 Department of Pediatrics, Center for Congenital Osteodystrophy – Sapienza University, Rome, Italy; 4 Department of Pediatrics, Sapienza University, Rome, Italy; 5 Department of Medical Genetics and Rare Orthopaedic Diseases, and CLIBI Laboratory, IRCCS Istituto Ortopedico Rizzoli, Bologna, Italy

**Keywords:** Genetic testing, Genetics research

## Abstract

Osteogenesis imperfecta (OI) is a rare genetic disorder of the connective tissue and 90% of cases are due to dominant mutations in *COL1A1* and *COL1A2* genes. To increase OI disease knowledge and contribute to patient follow-up management, a homogeneous Italian cohort of 364 subjects affected by OI types I–IV was evaluated. The study population was composed of 262 OI type I, 24 type II, 39 type III, and 39 type IV patients. Three hundred and nine subjects had a type I collagen affecting function mutations (230 in α1(I) and 79 in α2(I)); no disease-causing changes were noticed in 55 patients. Compared with previous genotype–phenotype OI correlation studies, additional observations arose: a new effect for α1- and α2-serine substitutions has been pointed out and heart defects, never considered before, resulted associated to quantitative mutations (*P* = 0.043). Moreover, some different findings emerged if compared with previous literature; especially, focusing the attention on the lethal form, no association with specific collagen regions was found and most of variants localized in the previously reported “lethal clusters” were causative of OI types I–IV. Some discrepancies have been highlighted also considering the “50–55 nucleotides rule,” as well as the relationship between specific collagen I mutated region and the presence of dentinogenesis imperfecta and/or blue sclera. Despite difficulties still present in defining clear rules to predict the clinical outcome in OI patients, this study provides new pieces for completing the puzzle, also thanks to the inclusion of clinical signs never considered before and to the large number of OI Italian patients.

## Introduction

Osteogenesis imperfecta (OI; MIM#166200, MIM#166210, MIM#259420, and MIM#166220) is a rare hereditary connective tissue disorder, with an incidence of ~1:15,000 to 1:20,000 in newborns [1]. The clinical spectrum, characterized by a wide intra- and interfamilial variability, is a continuum ranging from nearly asymptomatic individuals with occasional fractures, regular stature, and normal lifespan to phenotypes characterized by severe skeletal fragility, bone deformities, and significant growth deficiency up to neonatal lethality. Affected patients may also show a number of non-skeletal features such as blue sclera, hearing deficit, dentinogenesis imperfecta (DGI), cardiac defects, and joint hyperlaxity [2]. The disease is also characterized by a huge genetic heterogeneity, including autosomal-dominant, autosomal-recessive, and X-linked forms. About 90% of cases are caused by dominant mutations in *COL1A1* (MIM#120150) or *COL1A2* (MIM#120160) genes, encoding for α1(I) and α2(I) chains of type I collagen [3]. OI patients were distinguished in four clinical types according to clinical signs and radiographic features [4], then updated with more recent revisions [3]. Type I is the most common and includes patients with blue sclera and a range of fractures in the absence of bone deformities. OI type II results in prenatal or perinatal lethal outcome with severe deformities, congenital fractures, and marked deficiency of ossification. Type III is the most severe OI in non-lethal cases, characterized by a very short stature, long bone deformities, and multiple spinal problems. Lastly, type IV is a moderately deforming form with a variable phenotypic expression, with the presence of multiple fractures and white/gray sclera [3]. From a structural point of view, type I collagen is the major extracellular matrix protein of the bone, skin, tendon, and ligament. It is a heterotrimer constituted of two α1 chains and one α2-chain. Each chain has an amino- and a carboxyl-terminal propeptide at the ends and a central triple helical domain composed of 338 uninterrupted Gly-X-Y triplets. Glycine has a key role in collagen organization, as it is the only amino acid small enough to guarantee the proper folding of the polypeptide chains. The assembly of pro-α1 and pro-α2-chains into a triple helix begins at the carboxyl-terminal toward the amino-terminal end [5]. OI type I is usually caused by nonsense, frameshift, splice site mutations, and by a small number of big rearrangements that result in a reduced synthesis of structurally normal type I collagen, referred as a quantitative defect. On the other hand, OI types II–IV are mostly caused by substitutions of glycine in the triple helical domain, leading to abnormal type I collagen molecules that alter the triple helix folding and the fibrils formation, resulting in a qualitative defect [5, 6]. So far, more than 1500 *COL1A1-A2* distinct variants have been described in the OI Variant Database [7]. Despite this, some general rules about the genotype–phenotype relationship have been recognized [8–12], but all clinical manifestations have not been investigated yet and no directions about the wide clinical variability have been defined, including the different clinical outcome observed in patients who carry the same DNA change. In the present study, correlation between the occurrence of clinical manifestations and the type/location of genetic variants in *COL1A1-A2* genes was investigated to describe OI clinical spectrum in the Italian population and to obtain useful indications to better predict the evolution of the disease and the follow-up management.

## Subjects and methods

### Patients’ dataset

The study population included 364 patients (167 adults, 174 children, and 23 fetuses) from 295 unrelated families, evaluated between 2008 and 2016. The cutoff of 18 years was used to divide children and adults. In total, 249 patients were recruited at the Department of Medical Genetics, Istituto Ortopedico Rizzoli, Bologna (IOR), and 115 at the Department of Pediatrics “La Sapienza” University, Rome, both OI national reference center. Patients were clinically evaluated by multidisciplinary teams and classified by three clinicians with a long experience in OI, in accordance to the Van Dijk and Sillence criteria, and, in particular, to the described detailed clinical presentation and severity grading scale [3]. The study includes only individuals with a well-defined clinical diagnosis of OI types I–IV; patients with no extraskeletal involvement or with possible alternative diagnosis were excluded. All genetic, analyses were performed at the Department of Medical Genetics. Personal, familial, clinical and genetic data were collected and stored on an IT platform GephCard (Genotype-Phenotype Correlation, Analyses and Research Database) [13], in line with patient privacy rules, and legal and ethical data protection requirements. The study was approved by the Ethic Committee of IOR (ID 0024328/2014) in July 2014 and informed consent was obtained from all participants.

### Clinical features and genotype–phenotype correlation criteria

OI patients were evaluated for standing height, craniofacial signs (triangular face, frontal bossing, Wormian bones (WBs)), DGI, cardiac defects, scleral hue, skin problems, joint hyperlaxity, lumbar spine bone mineral density (BMD), and hearing loss. More in details, standing height was obtained by a stadiometer and only measured in lying down position for patients not able to stand up. All height measurements were recorded as means and SD in relation to age- and sex-specific Italian reference data (*Z*-score). A triangular face was defined as a triangular shaped face contour in frontal view and tapering/narrow chin; frontal bossing was described as bilateral bulging of the lateral frontal bone prominences with relative sparing of the midline [14]. The presence of WBs was classified as positive when the skull radiographs detected ten or more of them. DGI was considered as an abnormal structure of dentin and discoloration of teeth, ranging from gray–brown to blue opalescent. Cardiovascular abnormalities were detected by a cardiological evaluation in specialized centers. In particular, a complete transthoracic echocardiography was performed, including M-mode assessment of left ventricular dimensions and function, two-dimensional imaging, color flow mapping, and continuous and pulsed-wave Doppler, taking special care to the morphology and the function of the atrioventricular and semilunar valves. All detectable regurgitations were recorded and divided into mild, moderate, or severe [15], and only abnormalities from moderate to severe were included in the study and clustered together. Blue, gray, and white scleral hue were considered; patients were classified to have “blue sclera” if the color is Wedgewood blue [3], whereas they were categorized in “gray sclera” in case of blurred blue pale or gray. Skin abnormalities, as atypical scarring or easy bruising, were clinically evaluated by a specialist also in relation to the personal history; joint hyperlaxity was assessed by Beighton score and/or by history of multiple recurrent dislocations. According to a recent meta-analysis [16], bisphosphonate treatments were evaluated as not affecting other features in OI, except for BMD. To this, BMD from DXA (Dual X-ray Absorptometry) at the lumbar spine (L1–L4) was considered in children only, as data were collected before the treatment with anti-fractures drugs. Children were classified as affected by osteopenia for *Z*-score value included between − 1 and − 2, and affected by osteoporosis for value lower than − 2. To evaluate the presence of hearing loss, a pure tone audiometry was performed under standard conditions in a soundproof room; all patients with hearing impairment (conductive, sensorineural, and mixed) were clustered together. Variants were grouped in quantitative or qualitative defects, assuming that all nonsense, frameshift, large rearrangement, and splice site mutations would cause an haploinsufficiency, whereas glycine substitutions would lead to type I collagen qualitative alterations [5, 6].

### Type I collagen mutations analysis

For each proband, the screening of all coding exons of *COL1A1-A2* genes, including the exon–intron boundaries, was performed by qPCR with high-resolution melt (HRM) analysis, using SensiMix HRM kit (Bioline, London, UK) [5], able to simultaneously identify point mutations and large deletions/insertions. All amplicons with an abnormal profile were analyzed by Sanger sequencing (Thermo Fisher Scientific, Waltham, MA, USA). The sequences obtained were aligned with the GenBank reference sequences of *COL1A1* gene (NM_000088.3) and *COL1A2* (NM_000089.3). DNA alterations were recorded according to HGVS (Human Genome Variation Society) recommendations (http://varnomen.hgvs.org/). Patients with coding variants of unknown significance were excluded from the work. All DNA modifications included in this study were pathological and deposited into the OI Variant Database (https://oi.gene.le.ac.uk/home.php). In the text, the terms “mutations” and “affecting function alterations” were used to refer to a disease-causing change.

### Statistical analyses

The entire dataset was considered in all the analyses according to data availability. Statistical associations were also repeated considering children and adults separately to better evaluate potential age-related evolution. All continuous data were expressed in terms of the mean and the SD, the categorical data were expressed as frequency and percentages. The Kolmogorov–Smirnov test was performed to test normality of continuous variables. The analysis of variance (ANOVA) test was performed to assess the between-groups differences of continuous, normally distributed and homoscedastic data; the Mann–Whitney test was used otherwise. The ANOVA test followed by the Scheffè post hoc pairwise comparison was used also to assess the among-groups differences of continuous, normally distributed, and homoscedastic data; the Kruskal–Wallis test followed by the Mann–Whitney test with the Bonferroni correction for multiple comparison was used otherwise. Fisher’s *χ*^2^-test was performed to investigate the relationships between dichotomous variables. Pearson’s *χ*^2^-test, evaluated by exact methods for small samples, was performed to investigate the relationships between categorical variables. Spearman’s rank correlation was used to assess the relationship between continuous variables; Kendall tau correlation, evaluated by exact methods for small samples, was used to assess the correlation between ordinal variables. For all tests, *P* < 0.05 was considered significant. All statistical analyses were performed using SPSS v.19.0 (IBM, Corp., Armonk, NY, USA).

## Results

### Molecular genetics findings

Molecular screening identified a collagen mutation in 309 patients, 230 in α1(I) and 79 in α2(I) (Additional Files 1). Overall, 114 mutations lead to a qualitative defect (45 in α1, 69 in α2) and 195 to a quantitative alteration (185 in α1, 10 in α2). Considering the collagen structure, 33 subjects carried a mutation in the N-propeptide, 11 in the C-propeptide, and 265 in the triple helix domain. A total of 187 distinct mutations were identified: 143 distinct point mutations were localized on *COL1A1*, 42 on *COL1A2* gene, and 2 large rearrangements: a deletion of the first six exons of *COL1A1* and a partial deletion of 13–14 *COL1A2* exons. No mutations were found in 55 patients (15.1%), also tested through NGS panel to exclude the presence of recessive or X-linked OI forms.

### Clinical evaluation and demographic information

The study population consisted of 195 females, 156 males, and 13 fetuses of unknown gender. According to Van Dijk and Sillence classification [3], 262 patients were affected by OI type I (72%), 24 by type II (6.6%), 39 by type III (10.7%), and 39 by type IV (10.7%). The study cohort consisted of 174 children (mean age 7.27 ± 4.7 years), 167 adults (mean age 37 ± 12 years), and 23 fetuses. Data on disease inheritance were available for 274 patients: 158 (57.7%) have a positive family history, whereas 116 (42.3%) present a sporadic form. A positive family history is linked to OI type I and IV (detected in 64.8% and 66.7% of cases, respectively), whereas sporadic forms are related to OI type II and III (recorded in 77.8% and 81.5% of patients) (*P* < 0.0005); of note, the incidence of a positive familiarity decrease at the increasing of OI severity (Kendal tau = − 0.258, *P* < 0.0005).

### Genotype–phenotype correlation

**Fig. 1:**
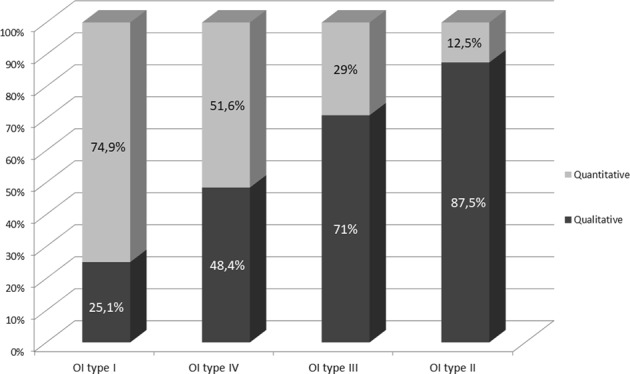
Relationship between OI types I–IV and the type of genetic mutation (qualitative or quantitative). The frequency has been reported for each class

**Fig. 2:**
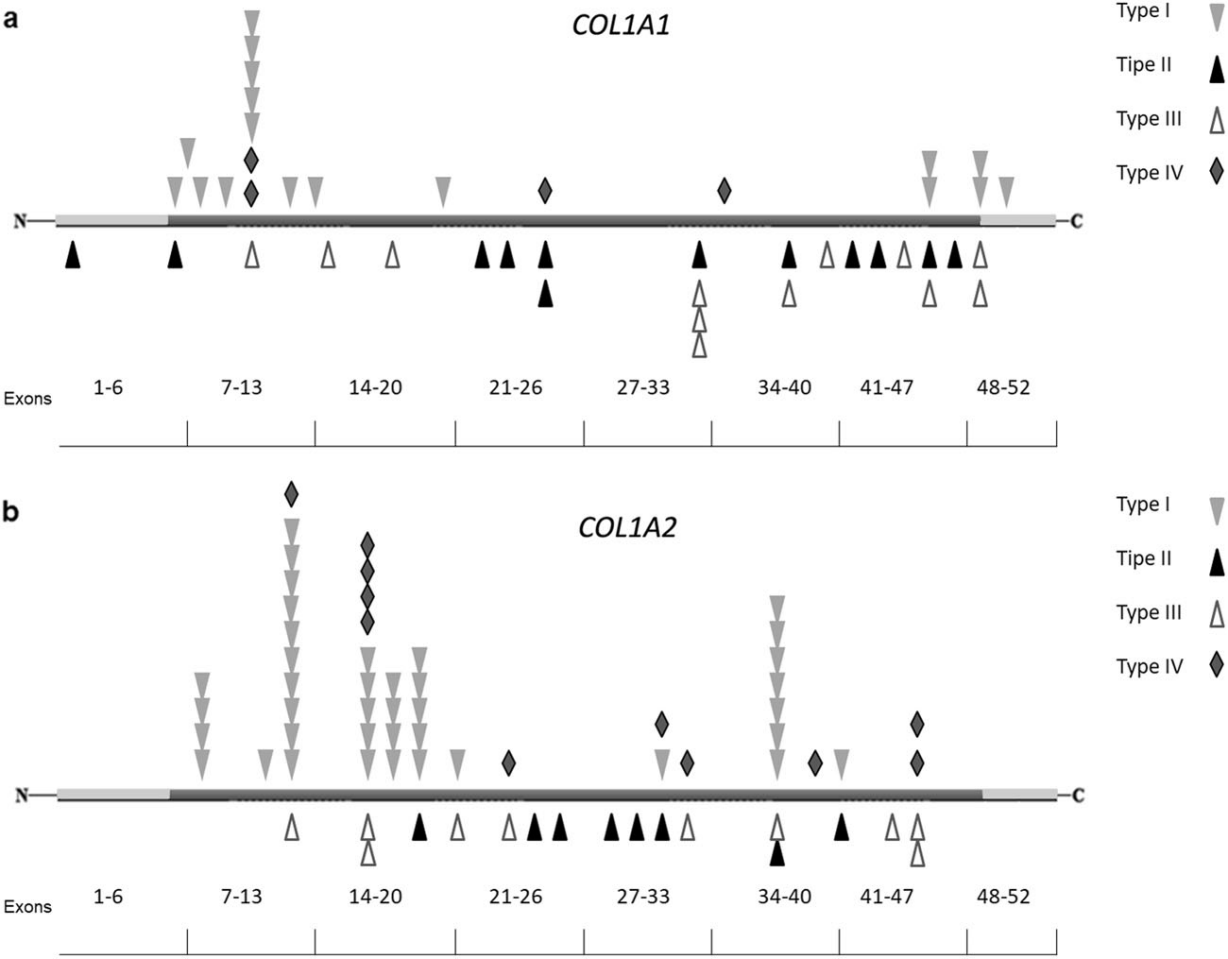
Glycine substitutions distribution along the α1- (**a**) and α2-chains (**b**) in relationship with the clinical outcome. Each symbol corresponds to a patient

The OI clinical types’ distribution, stratified on the gene involved, is described in Table 1. Concerning the 55 individuals with a clinical OI diagnosis and a negative screening result, 70.9% is OI type I, 14.55% OI type III, and 14.55% OI type IV. Quantitative alterations are mainly responsible for OI type I, whereas qualitative defects are significantly associated to OI types II–IV (Fig. 1; *P* < 0.0005), showing an ordinal and increasing correlation with the severity worsening of the disease (Kendall tau = − 0.25, *P* < 0.0005). Analyzing the collagen genes separately, the ordinal correlation was maintained only for *COL1A1* (*P* < 0.0005). Considering collagen I propeptides, mutations in the C-terminal domain are always responsible for a non-lethal disease, frequently causing a mild or moderate phenotype (90%). Mutations located in the *N*-propeptide cover the full spectrum of OI types, including an OI case with mild Ehlers–Danlos syndrome (EDS) signs, but resulting predominantly in OI type I and IV (93.9%). In the α1-C-propeptide, 6/11 cases (54.5%) had mutations located within 50–55 nucleotides upstream of the most 3′ exon–exon junctions or in the last exon of the gene (c.3653delC and c.3925C > T), not causing protein degradation, as previously reported [17, 18], but responsible of structural defects and severe disease forms. On the contrary, c.3807 G > A described inside this range is causative of mild phenotype in 2/2 cases, belonging to the same family. Similarly, c.3727 G > T was 50–55 nucleotides upstream the 3′ exon–exon junction but related to a moderate OI form. Finally, c.4332dupC, located in the last exon, was identified both in mild and severe OI cases. Concerning the α2-chain, the only mutation identified in the C-propeptide located in the above-mentioned domain is causative of mild OI. Considering clinical phenotype compared with location of glycine mutations, a severity gradient from N-terminal to C-terminal end was identified in both genes (Fig. 2). The majority of quantitative mutations related to OI type I was located in the first part of the α-helical domain (exons 7–14 for α1 and exons 8–17 for α2); glycine substitutions related to OI type II and III were distributed along the entire length of *COL1A1* gene, even if they were more frequent in the second part of the gene.

**Table 1:** Relationship between OI types and mutated genes

Clinical types	No. of patients with *COL1A1* mutations	No. of patients with *COL1A2* mutations	No. of patients without mutation in collagen I
OI type I	175 (66.8%)	48 (18.3%)	39 (14.9%)
OI type II	15 (62.5%)	9 (37.5%)	/
OI type III	20 (51.3%)	11 (28.2%)	8 (20.5%)
OI type IV	20 (51.3%)	11 (28.2%)	8 (20.5%)

OI: Osteogenesis Imperfecta

### Clinical variability

Different clinical outcomes in the presence of a same causative collagen mutation was evaluated for the 59 genetic alterations shared by more than one patient (45 in α1; 14 in α2). Ten out of 45 *COL1A1* mutations (5/7 qualitative and 5/38 quantitative defects) and 5 out of 14 *COL1A2* disease-causing changes (all qualitative defects) were related to clinical class variability (25.4%). In five glycine substitutions [α1-chain: p.(Gly491Ser); p.(Gly560Ser); p.(Gly767Ser); α2-chain: p.(Gly193Ser); p.(Gly874Asp)] a variability ranging from lethal to non-lethal forms was observed. Considering the intra-familial cases, a different outcome was observed in 9 out of 36 families (25%), with a disease worsening in the new generations in 4 of them [α1-chain: p.(Pro997Leufs*111); p.(Gly491Ser); α2-chain: p.(Gly193Ser); p.(Gly874Asp)].

### OI lethal form

Twenty-four out of 364 OI cases (6.6%) showed a lethal form. Specifically, 21 glycine substitutions and 3 different splice site mutations were detected (Table 2; 16 are novel). Considering the glycine substitution type, lethal outcomes are related to charged (Aspartic acid, Glutamic acid, Arginine) or branched (Valine) amino acids rather than nonpolar (Alanine) or neutral polar ones (Cysteine and Serine) (30.8% vs. 8.1%; *P* = 0.003). Analyzing the collagen genes separately, the correlation was maintained only for *COL1A2* gene (*P* = 0.01); 88.9% of lethal cases with *COL1A2* mutations is associated to substitutions by charged or branched amino acids. Concerning *COL1A1*, lethal mutations are distributed along the entire length of the α-helical domain, except for one localized in the N-terminal propeptide. Only one glycine mutation was detected in a previously reported “lethal cluster” inside the major ligand-binding region three (MLBR3) [8]; on the contrary, the non-lethal glycine substitution p.(Gly974Ala), identified in a OI type III patient, resided in the “lethal domain” inside the MLBR2 [8]. All *COL1A2* lethal mutations are localized in the helical region from exons 19 to 41. Only four of eight mutations were identified in one of the eight previously described “lethal domains” [8]. In contrast, six missense mutations [p.(Gly454Ala); p.(Gly649Asp); p.(Gly661Cys); p.(Gly661Asp); p.(Gly874Asp); p.(Gly991Val)] causative of non-lethal phenotypes were identified inside the domains 1, 3, and 7. In our dataset, the *COL1A2* second domain (range p.Gly541-Gly592) was the only confirmed as “lethal cluster.”

**Table 2:** Mutations involved in the lethal outcome

Gene	Exon	DNA change	Protein change
*COL1A1*	1	c.64 G > C	p.(Gly22Arg)
	8	c.608 G > A	p.(Gly203Asp)
	20	c.1353 + 1 G > A	/
	21	c.1462-2 A > G	/
	22	c.1471 G > A	p.(Gly491Ser)
	23	c.1562 G > A	p.(Gly521Glu)
	25	c.1678G > A	p.(Gly560Ser)
	25	c.1714G > C	p.(Gly572Arg)
	26	c.1821 + 4_1821 + 7del	/
	33_34	c.2299 G > A	p.(Gly767Ser)
	37	c.2515 G > C	p.(Gly839Arg)
	42	c.3038 G > A	p.(Gly1013Glu)
	43	c.3065 G > T	p.(Gly1022Val)
	45	c.3235 G > C	p.(Gly1079Arg)
	46	c.3263 G > C	**p.(Gly1088Ala)**
*COL1A2*	19	c.1027 G > A	p.(Gly343Arg)
	25	c.1460 G > A	p.(Gly487Gu)
	26	c.1541 G > T	p.(Gly514Val)
	29	c.1685G > A	**p.(Gly562Asp)**
	30	c.1730G > A	**p.(Gly577Asp)**
	31	c.1774G > A	**p.(Gly592Ser)**
	38	c.2333 G > A	p.(Gly778Asp)
	41	c.2621 G > A	**p.(Gly874Asp)**

The five mutations identified in the “lethal clusters” described by Marini et al. [8] are in bold

### OI patients with EDS signs

Our OI cohort was also evaluated for the presence of joint hypermobility and skin hyperelasticity with easy bruising and abnormal healing with atrophic or hypertrophic scars. These features were detected in five patients (1.4%) clinically diagnosed as OI, suggesting an OI/EDS overlap phenotype [19]. In all of these cases, a disease-causing change in collagen I was identified. An OI type IV subject carried the multi-exon deletion c.(?_−1)_(543 + 1_544–1)del in *COL1A1* gene. The other cases classified as OI type I have a mutation within the most N-terminal part of α2(I) helical domains: c.335 G > T p.(Gly112Val), c.432 + 1 G > A, and c.910 G > A p.(Gly304Ser).

### Stature evaluation

Standing height was evaluated in 291 OI patients (129 males and 162 females), 148 children, and 143 adults. Subjects with OI type I (*n* = 227) have a stature on average − 10,746 compared with − 25,062 for type IV (*n* = 34) and − 62,418 for type III (*n* = 30) (*P* < 0.0005), showing as the median height tends to significantly decrease with the increasing of disease severity (*P* < 0.0005). The relation between OI type III and short stature was also confirmed considering adults (*P* < 0.0005) and children (*P* = 0.001) separately. In addition, patients with qualitative defects are more associated to short stature than those with quantitative mutations (− 30,936 vs. − 13,183; *P* < 0.0005). The presence of a *COL1A2* mutation significantly influences the stature (*P* = 0.003); in particular, the pairwise comparison showed that subjects with *COL1A2* mutations are lower than those with no mutations (*P* = 0.002) or *COL1A1* mutations (*P* = 0.046) (Table 3 and Additional File 2).

**Table 3:** Clinical characteristics of our dataset subdivided by OI types

	OI type I	OI type II	OI type III	OI type IV	*p*-Value
Subjects	262	24	39	39	/
Gender M/F	117/145	6/5	18/21	15/24	NS
Familial/sporadic cases	64.8% (129)/ 35.2% (70)	22.2% (4)/ 77.8% (14)	18.5% (5)/ 81.5% (22)	66.7% (20)/ 33.3% (10)	*P* < 0.0005
Qualitative/quantitative	25.1%(56)/ 74.9% (167)	87.5% (21)/ 12.5% (3)	71% (22)/ 29% (9)	48.4% (15)/ 51.6% (16)	*P* < 0.0005
DGI %	16.8%	/	58.3%	44.8%	*P* < 0.0005
Height in *z*-score	− 10,746	/	− 62,418	− 25,062	*P* < 0.0005
Cardiac defects %	23.9%	/	19.0%	41.7%	NS
Blue sclera %	79.2%	/	54.3%	50.0%	*P* < 0.0005
Gray sclera %	9.6%	/	22.9%	31.6%	
WB %	13.8%	/	40.0%	42.1%	*P* < 0.0005
Triangular face %	7.6%	/	81.3%	21.7%	*P* < 0.0005
Frontal bossing %	12.4%	/	36.0%	21.4%	*P* = 0.008

DGI: Dentinogenesis Imperfecta, OI: Osteogenesis Imperfecta, NS: Not Significant, WB: Wormian Bones

### Craniofacial signs

A triangular face was detected in 19% of evaluated patients (25/131; 10 children and 15 adults). Our results show how it is a typical OI type III sign (81.3%), whereas it is scarcely associated with OI type IV (21.7%) and type I (7.6%) (*P* < 0.0005). *COL1A2* mutations seem to be related to the development of a triangular face (*P* = 0.057): 36% of subjects with α2-chain mutations developed a triangular face vs. 15.3% of those with α1-chain mutations and 14.3% of those with no mutation. Sixteen percent of evaluated patients has frontal bossing (37/231; 24 children and 13 adults): 36% of OI type III patients, 21.4% of OI type IV, and 12.4% of OI type I, associating this feature with OI type III (*P* = 0.008). WBs were detected in 20.9% of evaluated patients (38/182). As expected, this is significantly associated with OI type III and IV (40% and 42.1%, respectively), whereas type I patients result less inclined to present this feature (13.8%) (*P* < 0.0005; Table 3 and Additional File 2).

### Dentinogenesis imperfecta

The presence of DGI was detected in the 24.5% of evaluated patients (58/237), 18 adults, and 40 children. DGI was detected in 31/184 OI type I patients (16.8%), 14/24 OI type III (58.3%), and 13/29 OI type IV (44.8%), thus being related to OI type IV and III (*P* < 0.0005); in particular, the increasing of clinical severity is related to an ordinal increment of the number of patients with DGI (Kendall tau = 0.328, *P* < 0.0005). In 74.1% of patients with DGI (43/58), a collagen type I mutation was identified. Qualitative defects influence the insurgence of DGI much more than quantitative alterations (35.6% vs. 16.7%; *P* = 0.0068). In particular, 21/59 patients with a glycine substitution developed DGI, mostly characterized by a glycine to serine replacement (14/21). No patient carrying a glycine mutation in the first 127 amino acids of the α1(I) triple helical domain (range p.Gly178-p.Gly305) developed DGI, which is evident in 8/14 with pathological alterations beyond p.Gly305. In the α2(I) triple helical domain, 1/9 individuals carrying the mutation p.(Gly196Asp) within the first 121 amino acids (range p.Gly90-p.Gly211) showed DGI, vs. 12/28 patients with mutations beyond p.Gly211. All mutations related to dental status alterations (*n* = 34) were detected in the helical domain without specific hotspots, except for a nonsense in α1-N-propeptide and a frameshift mutation in α1-C-propeptide (Table 3, Additional File 2, and Fig. 4).

### Cardiac defects

**Fig. 3:**
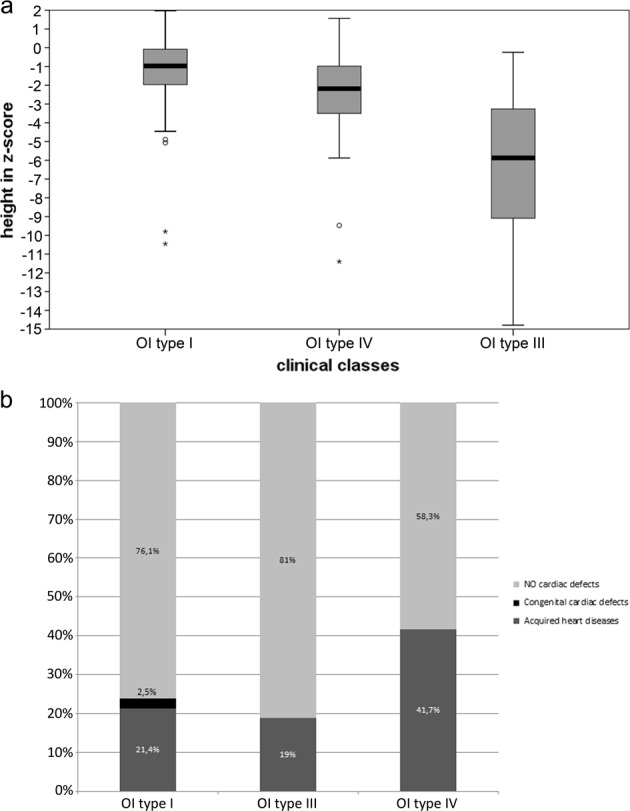
**a** Stature distribution in OI types I, III, and IV. **b** Relationship between OI clinical classes and the presence/absence of cardiac defects

The presence of a cardiac alteration was detected in 25.6% of evaluated cases (52/203; 21 males and 31 females). The group included 15 children and 37 adults. Heart problems were identified more frequently in the adult population (28.2% vs. 23.8%; *P* = 0.0015). In details, 48 patients (23.6%) had a valvulopathy and 4 individuals (1.97%) had other structural defects; specific cardiovascular diseases were detailed in Table 4. Considering valvulopathies the most frequent is mitral regurgitation, 40.4%, whereas the different types of structural defects are equally represented. Patients with type-IV OI showed a higher tendency to develop heart problems compared with other OI forms (Fig. 3b), even if no significant association was detect (*P* = 0.13). About the genetic background, cardiovascular abnormalities resulted more associated to quantitative alterations compared with structural ones (82.9% vs. 17.1%; *P* = 0.043). The same correlation was highlighted considering only the adult population (39.1% vs. 16.7%; *P* = 0.055). Mutations related to cardiac defects were found dispersed along the two genes, including propeptides’ regions (Table 3 and Additional File 2).

**Table 4:** Types of cardiovascular diseases

Cardiovascular diseases	No. of patients
Valvulopathies	Total 48
Mitral insufficiency	21
Tricuspid insufficiency	8
Aortic insufficiency	7
Pulmonary insufficiency	4
Mitral prolapse	3
Mitral + aortic insufficiency	2
Mitral + tricuspid insufficiency	2
Mitral + tricuspid + pulmonary insufficiency	1
Structural defects	Total 4
ASD	1
Tetralogy of Fallot	1
PDA	1
LVNC	1

ASD: Atrial Septal Defect, LVNC: Left Ventricular Non-Compaction, PDA: Patent Ductus Arteriosus

### Scleral hue

**Fig. 4:**
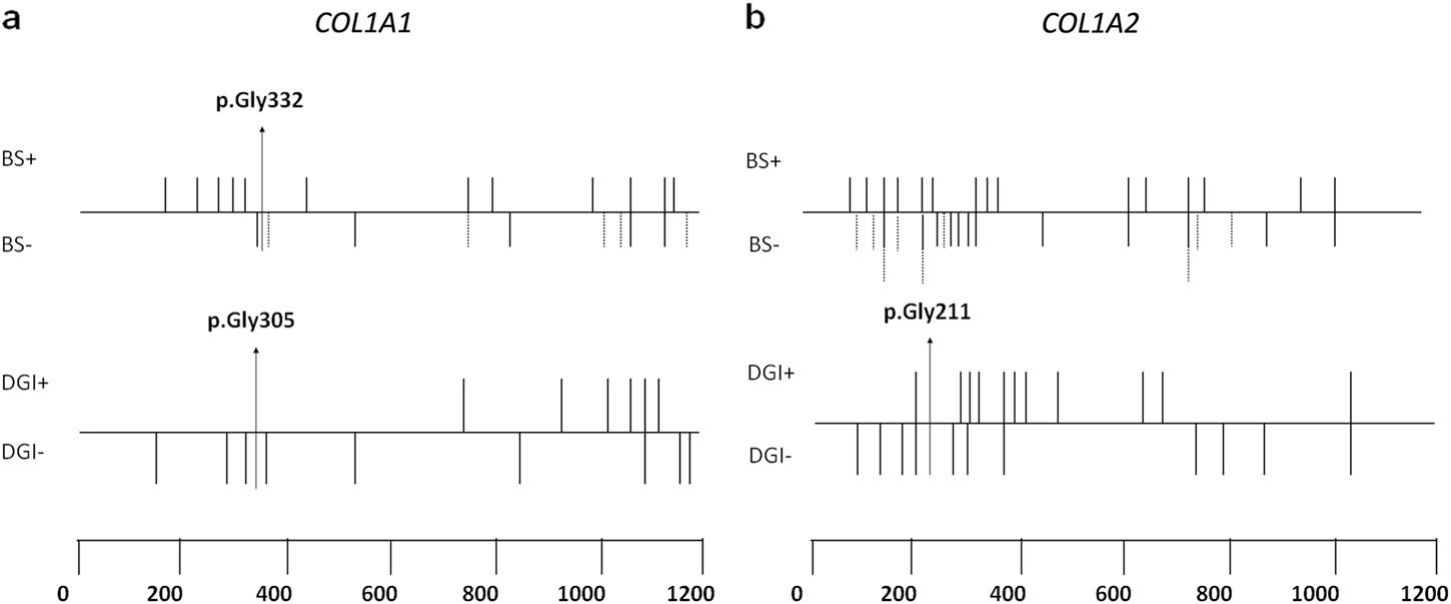
Dentinogenesis imperfecta (DGI) and scleral hue matching to the position of glycine mutations in *COL1A1-A2* genes. BS + = blue, BS − = gray/white; gray sclera is shown by dashed lines

Data on scleral hue were available for 334 patients, whereof 44 white, 245 blue, and 45 gray. The group included 171 children and 163 adults. The presence of blue sclera is clinically associated to OI type I (79.2%) and less represented in OI type IV and type III (50% and 54.3%, respectively); conversely, gray sclera is more frequent in OI type IV (31.6%) if compared with type I (9.6%) and type III (22.9%) (*P* < 0.0005). The same results emerged when evaluating adults and children separately (*P* < 0.0005). Qualitative alterations are associated to the presence of white and gray sclera, especially when the mutation is located in the α2(I) gene, whereas quantitative defects are related to blue sclera, specifically when located in the α1(I) gene (*P* < 0.0005). It is noteworthy that the absence of collagen mutations is associated to gray sclera (*p* < 0.0005). Concerning the α1(I) triple helical domain, glycine substitutions in the first 154 amino acids (range p.Gly178-p.Gly332) are related to blue sclera in 13/14 individuals, whereas mutations beyond p.Gly332 lead to colored sclera in 12/16 subjects, 7 blue and 5 gray (Table 3, Additional File 2, and Fig. 4).

### Other clinical features

Other clinical features, such as cutis laxa and/or sensitive skin, joint hypermobility, lumbar spine BMD, and hearing impairments, were investigated and the results are available in the Supplementary Materials (Additional File 3).

### Glycine substitutions in *COL1A1* and *COL1A2* genes

Assessing the different amino acid substitutions, glycine to serine was the most frequently observed (*n* = 51), followed by arginine (*n* = 24) and aspartic acid (*n* = 14). Considering the clinical effect, OI type II seems to be associated to Asp substitutions (*P* = 0.054), OI type III to α1-Ser substitutions, and OI type I to α2-Ser substitutions (*P* = 0.007).

## Discussion

The genotype–phenotype correlation study, performed by analyzing the largest Italian cohort ever considered, has allowed us to obtain precious information on 364 Italian patients with OI types I–IV analyzed for *COL1A1* and *COL1A2* genes. The width of the examined population permitted us to consider, for the first time, all OI clinical forms, therefore defining the distribution of Italian patients. The proportion of *COL1A1* and *COL1A2* mutations is in line with what already described in previous studies [11, 12], except for a light increase in number of patients without collagen I mutations (15.1% vs. 10%) [ref. 3, 6]. In particular, 14.9% of type I patients resulted negative for *COL1A1-A2* mutations. Considering that they were negative also for OI-related recessive and X-linked genes, and that patients with no extraskeletal involvement, with only bone fragility or with possible alternative diagnosis were excluded from the study, this suggests that other genes could be involved in the pathogenesis of the mildest form of the disease. A positive family history was present in 57.7% of patients, in line with 53% of cases reported in Korean population [20], but quite different to Chinese, Taiwanese, and another Italian population, characterized by a lower percentage (32–33%) of familial cases [11, 21, 22]. This difference could depend on the sample size and/or on population variances; other in-depth analysis would be necessary to clarify the diverse distributions. The percentage of patients with positive family history decreases with the increasing of phenotype severity, according to the hypothesis that the most severe cases related to collagen type I mutations are de novo [2]. In presence of a same mutation, the clinical classes variability occurs predominantly for qualitative defects. In details, the α2-chain mutations are causative of more variable outcomes than α1-chain mutations, most likely due to the greater number of glycine mutations identified on *COL1A2* gene. Moreover, only glycine substitutions showed outcomes ranging from mild to lethal.

We confirmed that quantitative defects are related to milder phenotypes, whereas qualitative alterations are responsible for the most severe forms [8, 11, 12]. In particular, an ordinal correlation between qualitative alterations frequency and disease worsening was shown for *COL1A1*, in line with the heterotrimeric structure of type I collagen, which requires two α1 chains and only one α2 chain, increasing the possibility that a qualitative α1 defect could alter a major number of collagen trimers and thus being responsible for a worse phenotype [23]. Nevertheless, the percentage of OI type I patients carrying a qualitative defect is halfway between previous findings: 25.1% in our study vs. 32% [ref. 12] and 9% [ref. 8]. Of note, no evident clinical differences beetwen OI type I patients carrying qualitative versus quantitative defects were observed. In line with recent studies [9, 12], it was demonstrated that α1-serine substitutions are related to OI type III and α2-serine substitutions to OI type I. Moreover, substitutions in the first 200 residues was confirmed to be usually non-lethal on both α1- and α2-chains. Previous studies defined the “50–55 nucleotides rule,” whereby premature termination codons located within the last 50–55 base pairs of most 3′’ exon–exon junction or in the last one of collagen genes do not generate non-mediated decay causing severe phenotypes [17, 24]. Symoens et al. [18] evaluated this rule in collagen I C-propeptide, proving its accuracy in 100% of cases. In our study, half of the cases follows the “50–55 nucleotides rule,” suggesting the need of further studies on a larger dataset to properly investigate this topic, as well as to highlight possible population-related genetic determinants that could influence this mechanism.

The severity pattern given by glycine substitutions in *COL1A1* gene showed an increase from the N-terminal to the C-terminal end, reflecting the assembly direction of collagen trimers [8]. This trend was confirmed for both genes; in particular, quantitative mutations located in the first part of the α-helical region are mostly causative of a mild form, whereas glycine substitutions in the second part of the genes are more often responsible for OI type II and III. It is noteworthy that a central zone virtually devoid of structural defects is present, where mutations probably go unnoticed as being responsible for too mild or too severe phenotypes.

Focusing the attention on lethal OI condition, all cases are related to glycine substitutions and splice-site mutations, proving that nonsense, frameshift mutations, and big rearrangements lead to surviving phenotype in 100% of cases. Differently from what was observed by Steiner et al. [2], no splice-site mutations beyond exon 25 in α2-chain are related to lethal outcomes, whereas just 7.7% beyond exon 14 of α1-chain lead to lethality. Lethal phenotypes are related to glycine substitutions with charged or branched amino acids, in agreement with Marini et al. [8]. Moreover, we proved a significant correlation between aspartate substitutions and OI type II. Referring to the previously reported “lethal clusters” [8], we confirmed only the range p.Gly541-Gly592 described in *COL1A2*. A glycine substitution in the other *COL1A1-A2* “lethal domains” was found in eight patients with no-lethal phenotypes, in line with Rauch et al. [9]. These observations, important for genetic counseling and prenatal diagnosis, suggest to better investigate the clusters function.

About height evaluation, OI type IV and III were correlated to shorter stature than type I, in agreement with literature [12]. This was confirmed both for adults and children, proving that this correlation is maintained independently from age [9]. From a molecular point of view, qualitative defects cause a more severe stature reduction, in line with other studies [9, 25] and with their worse effect. For the first time the frequency of patients with triangular face and frontal bossing in the Italian OI population was provided (19% and 16%, respectively). The WB percentage is far below than what was observed in other studies (20.9% vs. 58–59%) but with a similar distribution and correlation with the clinical types [26, 27]. Frontal bossing, WBs, and triangular face resulted to be associated to OI type III patients, also in the children group; this makes the presence of craniofacial signs a feature of severe forms. The DGI prevalence is in line with the existing literature [12, 28, 29], confirming the association with OI type IV and III, also in agreement with its relationship with qualitative mutations [11, 12, 28, 29]. Despite this, 16.7% of non-glycine mutations are related to the onset of DGI, an intermediate value if compared with Andersson et al. [29] (27%) and with that reported by Lund et al. [28] and Lindahl et al. [12] (4% and 1.4%, respectively). Lindahl et al. [12] observed that OI patients without collagen I mutations do not have dental alterations; on the contrary, in our dataset the 33.3% of this kind of patients develops DGI, thus associating this feature also to non-collagen OI types. Concerning missense mutations, the most frequent substitutions related to the presence of DGI regarded the serine, whereas none arginine substitution in the α1-chain leads to DGI, different from that identified by Rauch et al. [9]. Looking at the glycine substitutions in the first 127 amino acids of α1(I) and within the first 121 amino acids of α2(I) in the triple helical domain, we proved that mutations in these regions are related to DGI absence [9, 12], except for p.(Gly196Asp) in *COL1A2* gene.

About heart involvement, no previous genotype–phenotype correlation has been described, even if different studies highlighted that OI patients develop cardiovascular diseases more frequently than the general population [30–32]. According to our data, 23.6% of OI patients developed valvulopathy vs. the 2.5% of the industrialized countries population [33, 34]. Moreover, 19.7‰ of OI patients had other structural cardiac defects, compared with 6.82‰ of the overall Italian population [35]. These data confirm that alterations of collagen type I synthesis influence the risk of cardiovascular defects, especially valvulopathies [31], and for the first time a correlation with quantitative defects emerged. As they are usually related to milder OI clinical types, a wider sample size would be necessary to deeply understand this relationship. Even if more frequently found in the adults, cardiac alterations were also detected in children, showing a not concordant age of onset. In consideration of these observations, a recurrent follow-up would be advisable to early identify and to monitor the cardiological phenotype.

Concerning the scleral hue, no clear genetic rules have been yet defined; our data suggest that the presence of white and gray sclera is associated to qualitative alterations and to α2(I) mutations, whereas individuals with blue sclera are related to quantitative defects and to mutations in α1(I). This is in line with the mechanism potentially responsible for the blue sclera, whereby a quantitative reduction of type I collagen causes a thinner sclera, allowing to see the underlying choroidal veins. All patients, except one carrying a glycine substitution in the first 154 amino acids (p.Gly178-p.Gly332) of the α1(I) triple helical domain, have blue sclera, in line with what was previously observed [9, 12]. The association between this feature and clinical type has been confirmed in adults and children, demonstrating that even in adulthood the link remains significant despite the tendency to lose the scleral color with age increasing. Data collected about hearing loss, skin alterations, joint problems, and lumbar spine BMD did not reach any statistical significance, leaving open questions on these clinical signs.

In conclusion, the information outlined in this study gives a better overview of OI disease in a homogeneous Italian population. New evidences for genetic counseling have been highlighted, even if inter and intra-familial clinical variability remains one of the main OI critical issues to better investigate in the future. Taking advantage of the notable sample size, we also had the opportunity to evaluate some clinical signs never considered before. It would be desirable to invest in these population-related studies to corroborate the data so far observed and to extend the research field to other genetic and non-genetic determinants that could affect OI phenotype.

## Supplementary information

Additional file 1

Additional file 2

Additional file 3
