# Supplementary material for: Genotype–phenotype correlation study in 364 osteogenesis imperfecta Italian patients
Source: Eur J Hum Genet. 2019 Mar 18;27(7):1090–100. doi: 10.1038/s41431-019-0373-x (PMC6777444; doi:10.1038/s41431-019-0373-x)
Supplement: Supplementary file 1 — Additional file 1 [file 41431_2019_373_MOESM1_ESM.docx]

Additional file 1: COL1A1 disease-causing changes

| **LOVD Patient ID** | **Exon** | **DNA change** | **Type** | **Mutation effect** | **Protein** | **Legacy change** | **Type OI** |
| --- | --- | --- | --- | --- | --- | --- | --- |
| AN_004006 | 1-6 | c.(?_-1)_(543+1_544-1)del | Deletion | Multi-exon deletion | p.? | / | IV |
| AN_000079 | 1 | c.64G>C | Substitution | Missense | p.(Gly22Arg) | / | II |
| AN_004007 | 1i | c.103+1G>A | Substitution | Splice site | / | / | I |
| AN_004008 | 2 | c.120C>A | Substitution | Nonsense | p.Cys40* | / | I |
| AN_004009 | 2 | c.120C>A | Substitution | Nonsense | p.Cys40* | / | I |
| AN_004010 | 2 | c.158G>A | Substitution | Nonsense | p.Trp53* | / | IV |
| AN_004011 | 2 | c.158G>A | Substitution | Nonsense | p.Trp53* | / | IV |
| AN_006092 | 2 | c.189dupC | Duplication | Frameshift | p.(Asp64Argfs*9) | / | I |
| AN_006093 | 2 | c.189dupC | Duplication | Frameshift | p.(Asp64Argfs*9) | / | I |
| AN_006094 | 2 | c.189dupC | Duplication | Frameshift | p.(Asp64Argfs*9) | / | I |
| AN_000023 | 2 | c.199A>T | Substitution | Nonsense | p.(Lys67*) | / | I |
| AN_004012 | 2i | c.299-1G>C | Substitution | Splice site | / | / | I |
| AN_000030 | 2i | c.299-1G>C | Substitution | Splice site | / | / | I |
| AN_004013 | 2i | c.299_300del | Deletion | Frameshift | p.(Glu100Valfs*68) | / | I |
| AN_004014 | 4i | c.370-3_370-1delinsTC | Deletion/Insertion | Splice site | / | / | I |
| AN_004015 | 4i | c.370-3_370-1delinsTC | Deletion/Insertion | Splice site | / | / | I |
| AN_000054 | 5 | c.386delC | Deletion | Frameshift | p.(Pro129Leufs*136) | / | I |
| AN_004016 | 5 | c.386delC | Deletion | Frameshift | p.(Pro129Leufs*136) | / | I |
| AN_000005 | 5 | c.386dupC | Duplication | Frameshift | p.(Gly130Trpfs*39) | / | I |
| AN_000008 | 5 | c.386dupC | Duplication | Frameshift | p.(Gly130Trpfs*39) | / | I |
| AN_004017 | 5 | c.386dupC | Duplication | Frameshift | p.(Gly130Trpfs*39) | / | I |
| AN_004018 | 5 | c.386dupC | Duplication | Frameshift | p.(Gly130Trpfs*39) | / | I |
| AN_000002 | 5 | c.391C>T | Substitution | Nonsense | p.(Arg131*) | / | I |
| AN_004019 | 5 | c.410_413delAGCC | Deletion | Frameshift | p.Gln137Leufs*127 | / | I |
| AN_000084 | 5 | c.432dupC | Duplication | Frameshift | p.(Gly145Argfs*24) | / | I |
| AN_004020 | 5 | c.469G>T | Substitution | Nonsense | p.Gly157* | / | I |
| AN_004021 | 5 | c.469G>T | Substitution | Nonsense | p.Gly157* | / | I |
| AN_004022 | 5i | c.471+1G>C | Substitution | Splice site | / | / | I |
| AN_004023 | 6 | c.484C>T | Substitution | Nonsense | p.Gln162* | / | I |
| AN_000056 | 6 | c.506delA | Deletion | Frameshift | p.(Glu169Glyfs*96) | / | I |
| AN_004024 | 6 | c.506delA | Deletion | Frameshift | p.(Glu169Glyfs*96) | / | III |
| AN_004025 | 6 | c.506delA | Deletion | Frameshift | p.(Glu169Glyfs*96) | / | I |
| AN_004026 | 6i | c.543+1G>C | Substitution | Splice site | / | / | I |
| AN_000059 | 7 | c.578dupC | Duplication | Frameshift | p.(Gly194Trpfs*14) | / | I |
| AN_000006 | 7 | c.579delT | Deletion | Frameshift | p.(Gly194Valfs*71) | / | I |
| AN_001611 | 7 | c.579delT | Deletion | Frameshift | p.(Gly194Valfs*71) | / | IV |
| AN_001616 | 7 | c.579delT | Deletion | Frameshift | p.(Gly194Valfs*71) | / | I |
| AN_004003 | 7 | c.579delT | Deletion | Frameshift | p.(Gly194Valfs*71) | / | I |
| AN_004004 | 7 | c.579delT | Deletion | Frameshift | p.(Gly194Valfs*71) | / | I |
| AN_000087 | 7 | c.581G>C | Substitution | Missense | p.(Gly194Ala) | Gly16Ala | I |
| AN_004027 | 7 | c.581G>C | Substitution | Missense | p.(Gly194Ala) | Gly16Ala | I |
| AN_004028 | 7i | c.588+4A>T | Substitution | Splice site | / | / | I |
| AN_004029 | 8 | c.590G>C | Substitution | Missense | p.Gly197Ala | Gly19Ala | I |
| AN_004030 | 8 | c.608G>A | Substitution | Missense | p.Gly203Asp | Gly25Asp | II |
| AN_000039 | 9 | c.658delC | Deletion | Frameshift | p.(Arg220Glufs*45) | / | I |
| AN_000076 | 9 | c.658C>T | Substitution | Nonsense | p.(Arg220*) | Arg42* | I |
| AN_004031 | 9 | c.658C>T | Substitution | Nonsense | p.(Arg220*) | Arg42* | I |
| AN_000040 | 9 | c.662G>A | Substitution | Missense | p.(Gly221Asp) | Gly43Asp | I |
| AN_000088 | 9 | c.671delG | Deletion | Frameshift | p.(Gly224Valfs*41) | / | I |
| AN_004032 | 9 | c.671delG | Deletion | Frameshift | p.(Gly224Valfs*41) | / | I |
| AN_000085 | 9 | c.672_673delinsA | Deletion/Insertion | Frameshift | p.(Pro226Leufs*39) | / | I |
| AN_000046 | 9 | c.696+1G>A | Substitution | Splice site | / | / | I |
| AN_001612 | 10i | c.750+1G>A | Substitution | Splice site | / | / | III |
| AN_004033 | 10i | c.751-1G>C | Substitution | Splice site | / | / | I |
| AN_004034 | 10i | c.751-3_755del | Deletion | Splice site | / | / | I |
| AN_000020 | 11 | c.757C>T | Substitution | Nonsense | p.(Arg253*) | Arg75* | I |
| AN_000068 | 11 | c.757C>T | Substitution | Nonsense | p.(Arg253*) | Arg75* | IV |
| AN_004035 | 11 | c.757C>T | Substitution | Nonsense | p.(Arg253*) | Arg75* | IV |
| AN_004036 | 11 | c.757C>T | Substitution | Nonsense | p.(Arg253*) | Arg75* | IV |
| AN_004037 | 11 | c.757C>T | Substitution | Nonsense | p.(Arg253*) | Arg75* | III |
| AN_004038 | 11 | c.757C>T | Substitution | Nonsense | p.(Arg253*) | Arg75* | IV |
| AN_000043 | 11 | c.769G>A | Substitution | Missense | p.(Gly257Arg) | Gly79Arg | IV |
| AN_000086 | 11 | c.769G>A | Substitution | Missense | p.(Gly257Arg) | Gly79Arg | I |
| AN_000021 | 11 | c.769G>A | Substitution | Missense | p.(Gly257Arg) | Gly79Arg | I |
| AN_000064 | 11 | c.769G>A | Substitution | Missense | p.(Gly257Arg) | Gly79Arg | I |
| AN_004039 | 11 | c.769G>A | Substitution | Missense | p.(Gly257Arg) | Gly79Arg | IV |
| AN_004040 | 11 | c.769G>A | Substitution | Missense | p.(Gly257Arg) | Gly79Arg | I |
| AN_004041 | 11 | c.769G>A | Substitution | Missense | p.(Gly257Arg) | Gly79Arg | III |
| AN_004042 | 11 | c.769G>A | Substitution | Missense | p.(Gly257Arg) | Gly79Arg | I |
| AN_000045 | 11i | c.804+3_804+6del | Deletion | Splice site | / | / | I |
| AN_001602 | 12 | c.809dupT | Duplication | Frameshift | p.(Ser271Glnfs*16) | / | I |
| AN_004043 | 12 | c.809dupT | Duplication | Frameshift | p.(Ser271Glnfs*16) | / | I |
| AN_004044 | 12 | c.809dupT | Duplication | Frameshift | p.(Ser271Glnfs*16) | / | I |
| AN_004045 | 12 | c.809dupT | Duplication | Frameshift | p.(Ser271Glnfs*16) | / | I |
| AN_000099 | 13 | c.878G>T | Substitution | Missense | p.(Gly293Val) | Gly115Val | I |
| AN_000035 | 14 | c.913G>A | Substitution | Missense | p.(Gly305Ser) | Gly127Ser | I |
| AN_004005 | 14 | c.953delC | Deletion | Frameshift | p.Pro318Leufs*223 | / | IV |
| AN_004046 | 15 | c.994G>A | Substitution | Missense | p.(Gly332Arg) | Gly154Arg | III |
| AN_000052 | 15i | c.1003-2A>G | Substitution | Splice site | / | / | I |
| AN_004047 | 15i | c.1003-2A>G | Substitution | Splice site | / | / | I |
| AN_004048 | 15i | c.1003-2A>G | Substitution | Splice site | / | / | I |
| AN_004049 | 16i | c.1056+1G>A | Substitution | Splice site | / | / | III |
| AN_004050 | 17 | c.1057G>A | Substitution | Missense | p.(Gly353Ser) | Gly175Ser | III |
| AN_000010 | 17 | c.1099C>T | Substitution | Nonsense | p.(Gln367*) | Gln189* | I |
| AN_006001 | 17 | c.1128delT | Deletion | Frameshift | p.(Gly377Alafs*164) | / | I |
| AN_006002 | 17 | c.1128delT | Deletion | Frameshift | p.(Gly377Alafs*164) | / | I |
| AN_006003 | 17i | c.1155+1G>A | Substitution | Splice site | / | / | I |
| AN_006004 | 17i | c.1155+1G>A | Substitution | Splice site | / | / | I |
| AN_006005 | 17i | c.1155+1G>C | Substitution | Splice site | / | / | I |
| AN_000077 | 19 | c.1243C>T | Substitution | Nonsense | p.(Arg415*) | Arg237* | I |
| AN_006006 | 19 | c.1243C>T | Substitution | Nonsense | p.(Arg415*) | Arg237* | I |
| AN_006007 | 19 | c.1243C>T | Substitution | Nonsense | p.(Arg415*) | Arg237* | I |
| AN_006008 | 19 | c.1251delC | Deletion | Frameshift | p.Ser418Leufs*123 | / | I |
| AN_006009 | 19 | c.1251delC | Deletion | Frameshift | p.Ser418Leufs*123 | / | I |
| AN_000066 | 19i | c.1299+1G>A | Substitution | Splice site | / | / | I |
| AN_006010 | 19i | c.1299+1G>A | Substitution | Splice site | / | / | I |
| AN_006011 | 19i | c.1299+1G>A | Substitution | Splice site | / | / | I |
| AN_006012 | 19i | c.1299+1G>A | Substitution | Splice site | / | / | I |
| AN_006013 | 19i | c.1299+1G>A | Substitution | Splice site | / | / | I |
| AN_006014 | 19i | c.1299+1G>A | Substitution | Splice site | / | / | I |
| AN_006015 | 19i | c.1300-1G>A | Substitution | Splice site | / | / | I |
| AN_006016 | 20 | c.1310delG | Deletion | Frameshift | p.(Gly437Valfs*104) | / | I |
| AN_006017 | 20 | c.1316delC | Deletion | Frameshift | p.(Pro439Leufs*102) | / | I |
| AN_006018 | 20 | c.1316delC | Deletion | Frameshift | p.(Pro439Leufs*102) | / | I |
| AN_006019 | 20i | c.1353+1G>A | Substitution | Splice site | / | / | II |
| AN_001615 | 20i | c.1353+2T>G | Substitution | Splice site | / | / | I |
| AN_006020 | 21 | c.1380dupT | Deletion | Frameshift | p.(Gly461Trpfs*14) | / | I |
| AN_000072 | 21 | c.1414C>T | Substitution | Nonsense | p.(Arg472*) | Arg294* | I |
| AN_001618 | 21 | c.1414C>T | Substitution | Nonsense | p.(Arg472*) | Arg294* | I |
| AN_006021 | 21 | c.1414C>T | Substitution | Nonsense | p.(Arg472*) | Arg294* | I |
| AN_006022 | 21 | c.1414C>T | Substitution | Nonsense | p.(Arg472*) | Arg294* | I |
| AN_006023 | 21 | c.1414C>T | Substitution | Nonsense | p.(Arg472*) | Arg294* | I |
| AN_006024 | 21i | c.1461+1G>A | Substitution | Splice site | / | / | I |
| AN_000057 | 21i | c.1461+1G>C | Substitution | Splice site | / | / | I |
| AN_006025 | 21i | c.1462-2A>G | Substitution | Splice site | / | / | II |
| AN_000019 | 22 | c.1471G>A | Substitution | Missense | p.(Gly491Ser) | Gly313Ser | I |
| AN_006026 | 22 | c.1471G>A | Substitution | Missense | p.(Gly491Ser) | Gly313Ser | II |
| AN_006027 | 22 | c.1497_1498dupTG | Duplication | Frameshift | p.Gly500Valfs*42 | / | I |
| AN_006028 | 23 | c.1562G>A | Substitution | Missense | p.(Gly521Glu) | Gly343Glu | II |
| AN_006029 | 23 | c.1573delG | Deletion | Frameshift | p.(Glu525Lysfs*16) | / | I |
| AN_006030 | 23 | c.1573delG | Deletion | Frameshift | p.(Glu525Lysfs*16) | / | I |
| AN_006031 | 23 | c.1573delG | Deletion | Frameshift | p.(Glu525Lysfs*16) | / | I |
| AN_006032 | 24 | c.1667dupC | Duplication | Frameshift | p.(Gly557Trpfs*30) | / | IV |
| AN_006033 | 24 | c.1667delC | Deletion | Frameshift | p.(Pro556Leufs*24) | / | I |
| AN_006034 | 24 | c.1667delC | Deletion | Frameshift | p.(Pro556Leufs*24) | / | I |
| AN_001603 | 24i | c.1669-1G>C | Substitution | Splice site | / | / | IV |
| AN_000044 | 25 | c.1678G>A | Substitution | Missense | p.(Gly560Ser) | Gly382Ser | IV |
| AN_006035 | 25 | c.1678G>A | Substitution | Missense | p.(Gly560Ser) | Gly382Ser | II |
| AN_000038 | 25 | c.1714G>C | Substitution | Missense | p.(Gly572Arg) | Gly394Arg | II |
| AN_006036 | 25 | c.1719_1720insAC | Insertion | Frameshift | p.(Arg574Thrfs*7) | / | I |
| AN_000022 | 25 | c.1719_1720insAC | Insertion | Frameshift | p.(Arg574Thrfs*7) | / | I |
| AN_000095 | 25 | c.1719_1720insAC | Insertion | Frameshift | p.(Arg574Thrfs*7) | / | I |
| AN_000037 | 26 | c.1789G>T | Substitution | Nonsense | p.(Glu597*) | Glu419* | IV |
| AN_000092 | 26 | c.1792C>T | Substitution | Nonsense | p.(Arg598*) | Arg420* | I |
| AN_006037 | 26 | c.1792C>T | Substitution | Nonsense | p.(Arg598*) | Arg420* | I |
| AN_006038 | 26 | c.1804G>T | Substitution | Nonsense | p.(Gly602*) | Gly424* | I |
| AN_006039 | 26i | c.1821+1G>A | Substitution | Splice site | / | / | IV |
| AN_000049 | 26 | c.1821+1G>A | Substitution | Splice site | / | / | I |
| AN_000051 | 26 | c.1821+1G>A | Substitution | Splice site | / | / | I |
| AN_006040 | 26 | c.1821+1G>A | Substitution | Splice site | / | / | I |
| AN_006041 | 26 | c.1821+1G>A | Substitution | Splice site | / | / | I |
| AN_006042 | 26 | c.1821+1G>A | Substitution | Splice site | / | / | I |
| AN_006043 | 26i | c.1821+4_1821+7del | Deletion | Splice site | / | / | II |
| AN_006044 | 27 | c.1865delC | Deletion | Frameshift | p.(Pro622Leufs*144) | / | I |
| AN_006045 | 27i | c.1875+3G>T | Substitution | Splice site | / | / | I |
| AN_000058 | 28 | c.1920dupC | Duplication | Frameshift | p.(Gly641Argfs*14) | / | I |
| AN_000009 | 28i | c.1930-2A>C | Substitution | Splice site | / | / | I |
| AN_006046 | 28i | c.1930-2A>G | Substitution | Splice site | / | / | I |
| AN_000034 | 29i | c.1984-2A>G | Substitution | Splice site | / | / | I |
| AN_000097 | 31 | c.2037_2038del | Deletion | Frameshift | p.(Gly680Phefs*29) | / | I |
| AN_000027 | 31 | c.2086_2100delinsGCTGCAAGGTCCCCAT | Deletion/Insertion | Frameshift | p.(Pro696Alafs*14) | / | I |
| AN_000011 | 31 | c.2089C>T | Substitution | Nonsense | p.(Arg697*) | Arg519* | I |
| AN_006047 | 31 | c.2089C>T | Substitution | Nonsense | p.(Arg697*) | Arg519* | I |
| AN_000093 | 32 | c.2216delC | Deletion | Frameshift | p.(Pro739Glnfs*27) | / | IV |
| AN_006048 | 32i | c.2235_2235+1del | Deletion | Splice site | / | / | I |
| AN_000060 | 33_34 | c.2268_2269dup | Duplication | Frameshift | p.(Pro757Leufs*10) | / | I |
| AN_006049 | 33_34 | c.2268_2269dup | Duplication | Frameshift | p.(Pro757Leufs*10) | / | III |
| AN_006050 | 33_34 | c.2270delC | Deletion | Frameshift | p.(Pro757Leufs*9) | / | I |
| AN_000089 | 33_34 | c.2299G>A | Substitution | Missense | p.(Gly767Ser) | Gly595Ser | III |
| AN_000024 | 33_34 | c.2299G>A | Substitution | Missense | p.(Gly767Ser) | Gly589Ser | III |
| AN_006051 | 33_34 | c.2299G>A | Substitution | Missense | p.(Gly767Ser) | Gly589Ser | III |
| AN_006052 | 33_34 | c.2299G>A | Substitution | Missense | p.(Gly767Ser) | Gly589Ser | II |
| AN_000063 | 35 | c.2362_2384del | Deletion | Frameshift | p.(Gly788Serfs*9) | / | I |
| AN_006053 | 35 | c.2362G>A | Substitution | Missense | p.(Gly788Ser) | Gly610Ser | IV |
| AN_006054 | 36 | c.2398G>T | Substitution | Nonsense | p.(Gly800*) | Gly622* | I |
| AN_006055 | 36 | c.2398G>T | Substitution | Nonsense | p.(Gly800*) | Gly622* | I |
| AN_006056 | 36 | c.2398G>T | Substitution | Nonsense | p.(Gly800*) | Gly622* | I |
| AN_006057 | 36i | c.2451+1G>A | Substitution | Splice site | / | / | I |
| AN_000026 | 37 | c.2461G>A | Substitution | Missense | p.(Gly821Ser) | Gly643Ser | III |
| AN_004003 | 37 | c.2515G>C | Substitution | Missense | p.(Gly839Arg) | Gly661Arg | II |
| AN_001610 | 37 | c.2523delT | Deletion | Frameshift | p.(Gly842Alafs*266) | / | I |
| AN_000053 | 37 | c.2550delT | Deletion | Frameshift | p.(Gly851Alafs*257) | / | I |
| AN_000067 | 38 | c.2594dupG | Duplication | Frameshift | p.Gly866Argfs*47 | / | I |
| AN_000001 | 38 | c.2612delC | Deletion | Frameshift | p.(Pro871Leufs*237) | / | I |
| AN_006058 | 38 | c.2612_2613del | Deletion | Frameshift | p.(Pro871Argfs*41) | / | I |
| AN_001601 | 38 | c.2612dup | Duplication | Frameshift | p.(Gly872Trpfs*41) | / | I |
| AN_006059 | 38i | c.2613+6T>C | Substitution | Splice site | / | / | III |
| AN_001609 | 39 | c.2644C>T | Substitution | Nonsense | p.(Arg882*) | Arg704* | I |
| AN_000069 | 39i | c.2668-1G>T | Substitution | Splice site | / | / | I |
| AN_006060 | 40 | c.2684dup | Duplication | Frameshift | p.Gly896Trpfs*17 | / | I |
| AN_006061 | 40 | c.2684dup | Duplication | Frameshift | p.Gly896Trpfs*17 | / | I |
| AN_006062 | 40i | c.2829+1G>A | Substitution | Splice site | / | / | I |
| AN_006063 | 40i | c.2829+1G>A | Substitution | Splice site | / | / | I |
| AN_006064 | 41 | c.2870_2873del | Deletion | Frameshift | p.(Gln957Leufs*150) | / | I |
| AN_006065 | 41 | c.2921G>C | Substitution | Missense | p.(Gly974Ala) | Gly796Ala | III |
| AN_006066 | 41 | c.2935delT | Deletion | Frameshift | p.(Ser979Leufs*129) | / | IV |
| AN_000015 | 42 | c.2953C>T | Substitution | Nonsense | p.(Gln985*) | Gln807* | I |
| AN_006067 | 42 | c.2985delT | Deletion | Frameshift | p.(Pro997Leufs*111) | / | I |
| AN_006068 | 42 | c.2990delC | Deletion | Frameshift | p.(Pro997Leufs*111) | / | III |
| AN_006069 | 42 | c.2990delC | Deletion | Frameshift | p.(Pro997Leufs*111) | / | IV |
| AN_006070 | 42 | c.2991delT | Deletion | Frameshift | p.(Gly998Valfs*110) | / | I |
| AN_000078 | 42 | c.2991delT | Deletion | Frameshift | p.(Gly998Valfs*110) | / | I |
| AN_000090 | 42 | c.3008delC | Deletion | Frameshift | p.(Pro1003Leufs*105) | / | I |
| AN_006071 | 42 | c.3008delC | Deletion | Frameshift | p.(Pro1003Leufs*105) | / | I |
| AN_006072 | 42 | c.3008dupC | Duplication | Frameshift | p.(Gly1004Trpfs*8) | / | I |
| AN_006073 | 42 | c.3038G>A | Substitution | Missense | p.(Gly1013Glu) | / | II |
| AN_000012 | 43 | c.3065G>T | Substitution | Missense | p.(Gly1022Val) | Gly844Val | II |
| AN_006074 | 43 | c.3070_3071delinsG | Deletion/Insertion | Frameshift | p.(Pro1024Valfs*84) | / | I |
| AN_000080 | 43 | c.3076C>T | Substitution | Nonsense | p.(Arg1026*) | Arg848* | I |
| AN_006075 | 43 | c.3076C>T | Substitution | Nonsense | p.(Arg1026*) | Arg848* | I |
| AN_000098 | 44 | c.3118G>A | Substitution | Missense | p.(Gly1040Ser) | Gly862Ser | III |
| AN_004001 | 44 | c.3162delT | Deletion | Frameshift | p.(Gly1055Alafs*53) | / | I |
| AN_000061 | 44 | c.3168dupC | Duplication | Frameshift | p.Val1057Argfs*9 | / | I |
| AN_006076 | 44i | c.3207+1G>T | Substitution | Splice site | / | / | I |
| AN_006077 | 45 | c.3226G>A | Substitution | Missense | p.(Gly1076Ser) | Gly898Ser | III |
| AN_001613 | 45 | c.3235G>C | Substitution | Missense | p.(Gly1079Arg) | Gly901Arg | II |
| AN_006078 | 45 | c.3235G>A | Substitution | Missense | p.(Gly1079Ser) | Gly901Ser | I |
| AN_006079 | 45 | c.3235G>A | Substitution | Missense | p.(Gly1079Ser) | Gly901Ser | I |
| AN_001608 | 46 | c.3263G>C | Substitution | Missense | p.(Gly1088Ala) | Gly910Ala | II |
| AN_001619 | 46 | c.3277del | Deletion | Frameshift | p.(Arg1093Valfs*15) |  | I |
| AN_006080 | 46 | c.3359_3360+23del | Substitution | Splice site | / | / | I |
| AN_000014 | 46i | c.3369+1G>A | Substitution | Splice site | / | / | I |
| AN_006081 | 47 | c.3421C>T | Substitution | Nonsense | p.(Arg1141*) | Arg963* | I |
| AN_006082 | 47 | c.3421C>T | Substitution | Nonsense | p.(Arg1141*) | Arg963* | I |
| AN_000065 | 48 | c.3505G>A | Substitution | Missense | p.(Gly1169Ser) | Gly991Ser | I |
| AN_006083 | 48 | c.3505G>A | Substitution | Missense | p.(Gly1169Ser) | Gly991Ser | III |
| AN_000055 | 48 | c.3523G>T | Substitution | Missense | p.Gly1175Cys | Gly997Cys | III |
| AN_006084 | 48 | c.3524G>C | Substitution | Missense | p.(Gly1175Ala) | Gly997Ala | I |
| AN_006085 | 48i | c.3531+5G>A | Substitution | Splice site | / | / | I |
| AN_000018 | 49 | c.3569G>A | Substitution | Missense | p.Gly1190Asp | Gly1012Asp | I |
| AN_000017 | 49 | c.3653delC | Deletion | Frameshift | p.Ala1218Valfs*21 | / | I |
| AN_006086 | 49 | c.3727G>T | Substitution | Nonsense | p.(Glu1243*) | Gly1065* | IV |
| AN_000029 | 49 | c.3807G>A | Substitution | Nonsense | p.(Trp1269*) | Trp1091* | I |
| AN_006087 | 49 | c.3807G>A | Substitution | Nonsense | p.(Trp1269*) | Trp1091* | I |
| AN_000081 | 50 | c.3925C>T | Substitution | Nonsense | p.(Gln1309*) | Gln1131* | I |
| AN_006088 | 50 | c.3925C>T | Substitution | Nonsense | p.(Gln1309*) | Gln1131* | I |
| AN_006089 | 50 | c.3925C>T | Substitution | Nonsense | p.(Gln1309*) | Gln1131* | I |
| AN_006090 | 50 | c.3925C>T | Substitution | Nonsense | p.(Gln1309*) | Gln1131* | I |
| AN_004002 | 52 | c.4332dupC | Insertion | Frameshift | p.Asp1446Glyfs*105 | / | III |
| AN_006091 | 52 | c.4332dupC | Insertion | Frameshift | p.Asp1446Glyfs*105 | / | I |

COL1A2 disease-causing changes

| **LOVD Patient ID** | **Exon** | **DNA change** | **Type** | **Mutation effect** | **Protein** | **Legacy change** | **Type OI** |
| --- | --- | --- | --- | --- | --- | --- | --- |
| AN_000073 | 8 | c.335G>T | Substitution | Missense | p.(Gly112Val) | Gly22Val | I |
| AN_004005 | 8 | c.335G>T | Substitution | Missense | p.(Gly112Val) | Gly22Val | I |
| AN_004051 | 8 | c.335G>T | Substitution | Missense | p.(Gly112Val) | Gly22Val | I |
| AN_004052 | 8 | c.335G>T | Substitution | Missense | p.(Gly112Val) | Gly22Val | I |
| AN_000025 | 9i | c.432+1G>A | Substitution | Splice site | / | / | I |
| AN_004053 | 9i | c.432+1G>A | Substitution | Splice site | / | / | I |
| AN_000031 | 11 | c.506G>A | Substitution | Missense | p.(Gly169Glu) | Gly79Glu | I |
| AN_004054 | 12 | c.560G>A | Substitution | Missense | p.(Gly187Glu) | Gly97Glu | I |
| AN_004055 | 12 | c.569G>C | Substitution | Missense | p.(Gly190Ala) | Gly100Ala | IV |
| AN_000016 | 12 | c.577G>C | Substitution | Missense | p.(Gly193Arg) | Gly103Arg | I |
| AN_000047 | 12 | c.577G>A | Substitution | Missense | p.(Gly193Ser) | Gly103Ser | I |
| AN_004056 | 12 | c.577G>A | Substitution | Missense | p.(Gly193Ser) | Gly103Ser | I |
| AN_004057 | 12 | c.577G>A | Substitution | Missense | p.(Gly193Ser) | Gly103Ser | III |
| AN_000070 | 12 | c.577G>A | Substitution | Missense | p.(Gly193Ser) | Gly103Ser | I |
| AN_001606 | 12 | c.577G>A | Substitution | Missense | p.(Gly193Ser) | Gly103Ser | I |
| AN_004058 | 12 | c.577G>A | Substitution | Missense | p.(Gly193Ser) | Gly103Ser | I |
| AN_004059 | 12 | c.577G>A | Substitution | Missense | p.(Gly193Ser) | Gly103Ser | I |
| AN_001605 | 12 | c.587G>A | Substitution | Missense | p.(Gly196Asp) | Gly106Ser | I |
| AN_004060 | 12 | c.587G>A | Substitution | Missense | p.(Gly196Asp) | Gly106Ser | I |
| AN_004061 | 13-14 | c.(594+59_595-23)_(693+12_694-47)del | Deletion | Multi-exon deletion | / | / | III |
| AN_004062 | 14i | c.693+1G>A | Substitution | Splice site | / | / | I |
| AN_000003 | 17 | c.811G>C | Substitution | Missense | p.(Gly271Arg) | Gly181Arg | I |
| AN_004063 | 17 | c.811G>C | Substitution | Missense | p.(Gly271Arg) | Gly181Arg | I |
| AN_004064 | 17 | c.811G>C | Substitution | Missense | p.(Gly271Arg) | Gly181Arg | I |
| AN_004065 | 17 | c.811G>C | Substitution | Missense | p.(Gly271Arg) | Gly181Arg | I |
| AN_000075 | 17 | c.811G>C | Substitution | Missense | p.(Gly271Arg) | Gly181Arg | IV |
| AN_004066 | 17 | c.811G>C | Substitution | Missense | p.(Gly271Arg) | Gly181Arg | IV |
| AN_000083 | 17 | c.829G>C | Substitution | Missense | p.(Gly277Arg) | Gly187Arg | III |
| AN_000033 | 17 | c.829G>C | Substitution | Missense | p.(Gly277Arg) | p.Gly277Arg | IV |
| AN_000094 | 17 | c.838G>A | Substitution | Missense | p.(Gly280Ser) | Gly190Ser | I |
| AN_004067 | 17 | c.856G>A | Substitution | Missense | p.(Gly286Ser) | Gly196Ser | III |
| AN_000048 | 17 | c.865G>A | Substitution | Missense | p.(Gly289Ser) | Gly199Ser | IV |
| AN_004068 | 18 | c.910G>A | Substitution | Missense | p.(Gly304Ser) | Gly214Ser | I |
| AN_004069 | 18 | c.910G>A | Substitution | Missense | p.(Gly304Ser) | Gly214Ser | I |
| AN_004070 | 18 | c.910G>A | Substitution | Missense | p.(Gly304Ser) | Gly214Ser | I |
| AN_004071 | 18 | c.910G>A | Substitution | Missense | p.(Gly304Ser) | Gly214Ser | I |
| AN_001614 | 19 | c.946G>A | Substitution | Missense | p.(Gly316Ser) | Gly226Ser | I |
| AN_004072 | 19 | c.946G>A | Substitution | Missense | p.(Gly316Ser) | Gly226Ser | I |
| AN_001621 | 19 | c.1009G>A | Substitution | Missense | p.(Gly337Ser) | Gly247Ser | IV |
| AN_004073 | 19 | c.1009G>A | Substitution | Missense | p.(Gly337Ser) | Gly247Ser | I |
| AN_004074 | 19 | c.1009G>A | Substitution | Missense | p.(Gly337Ser) | Gly247Ser | I |
| AN_004075 | 19 | c.1027G>A | Substitution | Missense | p.(Gly343Arg) | Gly253Arg | II |
| AN_004076 | 19i | c.1036-1G>C | Substitution | Splice site | / | / | I |
| AN_000082 | 19i | c.1036-1G>C | Substitution | Splice site | / | / | I |
| AN_001607 | 21 | c.1153G>A | Substitution | Missense | p.(Gly385Arg) | Gly295Arg | III |
| AN_000007 | 21 | c.1171G>A | Substitution | Missense | p.(Gly391Ser) | Gly301Ser | I |
| AN_000050 | 21 | c.1197+5G>A | Substitution | Splice site | / | / | OI |
| AN_000032 | 24 | c.1361G>C | Substitution | Missense | p.Gly454Ala | Gly364Ala | IV |
| AN_000071 | 24 | c.1378G>A | Substitution | Missense | p.(Gly460Ser) | Gly370Ser | III |
| AN_004076 | 25 | c.1460G>A | Substitution | Missense | p.(Gly487Glu) | Gly397Glu | II |
| AN_004004 | 26 | c.1541G>T | Substitution | Missense | p.(Gly514Val) | Gly424Val | II |
| AN_004077 | 29 | c.1685G>A | Substitution | Missense | p.(Gly562Asp) | Gly472Asp | II |
| AN_004078 | 30 | c.1730G>A | Substitution | Missense | p.(Gly577Asp) | Gly487Asp | II |
| AN_004079 | 31 | c.1774G>A | Substitution | Missense | p.Gly592Ser | Gly502Ser | II |
| AN_004080 | 32 | c.1946G>A | Substitution | Missense | p.(Gly649Asp) | Gly559Asp | IV |
| AN_004081 | 32 | c.1946G>A | Substitution | Missense | p.(Gly649Asp) | Gly559Asp | I |
| AN_004082 | 33 | c.1981G>T | Substitution | Missense | p.(Gly661Cys) | Gly571Cys | IV |
| AN_004083 | 33 | c.1982G>A | Substitution | Missense | p.Gly661Cys | Gly571Asp | III |
| AN_000041 | 38 | c.2314G>A | Substitution | Missense | p.(Gly772Ser) | Gly682Ser | I |
| AN_004084 | 38 | c.2314G>A | Substitution | Missense | p.(Gly772Ser) | Gly682Ser | I |
| AN_000096 | 38 | c.2314G>A | Substitution | Missense | p.(Gly772Ser) | Gly682Ser | I |
| AN_004085 | 38 | c.2314G>A | Substitution | Missense | p.(Gly772Ser) | Gly682Ser | I |
| AN_004086 | 38 | c.2314G>A | Substitution | Missense | p.(Gly772Ser) | Gly682Ser | I |
| AN_004087 | 38 | c.2314G>A | Substitution | Missense | p.(Gly772Ser) | Gly682Ser | I |
| AN_004088 | 38 | c.2314G>A | Substitution | Missense | p.(Gly772Ser) | Gly682Ser | I |
| AN_004089 | 38 | c.2324G>A | Substitution | Missense | p.(Gly775Glu) | Gly685Glu | III |
| AN_004090 | 38 | c.2333G>A | Substitution | Missense | p.(Gly778Asp) | Gly688Asp | II |
| AN_000028 | 40 | c.2432G>C | Substitution | Missense | p.Gly811Ala | Gly721Ala | IV |
| AN_001604 | 41 | c.2599_2601del | Deletion | Frameshift | p.(Leu867del) | / | I |
| AN_000062 | 41 | c.2621G>A | Substitution | Missense | p.Gly874Asp | Gly784Asp | II |
| AN_004091 | 41 | c.2621G>A | Substitution | Missense | p.Gly874Asp | Gly784Asp | I |
| AN_004092 | 41 | c.2621G>A | Substitution | Missense | p.Gly874Asp | Gly784Asp | II |
| AN_004093 | 43i | c.2835+1G>A | Substitution | Splice site | / | / | I |
| AN_000036 | 45 | c.2972G>T | Substitution | Missense | p.(Gly991Val) | Gly901Val | III |
| AN_000013 | 46 | c.3034G>A | Substitution | Missense | p.(Gly1012Ser) | Gly922Ser | III |
| AN_004094 | 46 | c.3034G>A | Substitution | Missense | p.(Gly1012Ser) | Gly922Ser | IV |
| AN_004095 | 46 | c.3034G>A | Substitution | Missense | p.(Gly1012Ser) | Gly922Ser | III |
| AN_004096 | 46 | c.3034G>A | Substitution | Missense | p.(Gly1012Ser) | Gly922Ser | IV |
| AN_001617 | 51 | c.3897_3916del | Deletion | Frameshift | p.(Leu1300Glnfs*13) | / | I |
