# Supplementary material for: Genotype–phenotype correlation study in 364 osteogenesis imperfecta Italian patients
Source: Eur J Hum Genet. 2019 Mar 18;27(7):1090–100. doi: 10.1038/s41431-019-0373-x (PMC6777444; doi:10.1038/s41431-019-0373-x)
Supplement: Supplementary file 2 — Additional file 2 [file 41431_2019_373_MOESM2_ESM.docx]

**Additional file 2.** Summary tables with demographic and genetic data and clinical distribution for each OI phenotypic characteristic

|  |  | STATURE EVALUATION |  |
| --- | --- | --- | --- |
|  |  | Height in z-score | p-value |
| gender | male | -1.4818 (130) | P=0.043 |
|  | female | -2.0333 (161) |  |
| familiarity | yes | -1.5047 (130) | NS |
|  | no | -1.9177 (81) |  |
| mutated gene | COL1A1 | -1.6547 | P<0.0005 |
|  | COL1A2 | -2.6821 |  |
|  | no col1 mutations | -1.3830 |  |
| mutation type | qualitative | -3.0936 (80) | P<0.0005 |
|  | quantitative | -1.3183 (162) |  |
| clinical classes | OI type I | (227) | P<0.0005 |
|  | OI type III | (30) |  |
|  | OI type IV | (34) |  |

Data on stature evaluated on 291 patients

|  |  | WORMIAN BONES | |  |
| --- | --- | --- | --- | --- |
|  |  | yes | no | p-value |
|  | individuals | 38 | 144 | / |
| gender | male | 20% (17) | 80% (68) | NS |
|  | female | 21.4% (21) | 78.6% (77) |  |
| familiarity | yes | 19.4% (14) | 80.6% (58) | NS |
|  | no | 25.5% (12) | 74.5% (35) |  |
| mutated gene | COL1A1 | 29.3% (25) | 79.7% (98) | NS |
|  | COL1A2 | 22.2% (6) | 77.8% (21) |  |
|  | no col1 mutations | 21.2% (7) | 78.8% (26) |  |
| mutation type | qualitative | 25.6% (11) | 74.4% (32) | NS |
|  | quantitative | 18.7% (20) | 81.3% (87) |  |
| clinical classes | OI type I | 13.8% (19) | 86.2% (119) | P<0.0005 |
|  | OI type III | 40% (10) | 60% (15) |  |
|  | OI type IV | 42.1% (8) | 57.9% (11) |  |

Distribution of Wormian bones evaluated on 182 patients

|  |  | TRIANGULAR FACE | |  |
| --- | --- | --- | --- | --- |
|  |  | yes | no | p-value |
|  | individuals | 25 | 106 | / |
| gender | male | 15.9% (10) | 84.1% (53) | NS |
|  | female | 22.1% (15) | 77.9% (53) |  |
| familiarity | yes | 13.5% (7) | 86.5% (45) | NS |
|  | no | 35% (7) | 65% (13) |  |
| mutated gene | COL1A1 | 84.7% (72) | 15.3% (13) | P=0.057 |
|  | COL1A2 | 64% (16) | 36% (9) |  |
|  | no col1 mutations | 85.7% (18) | 14.3% (3) |  |
| mutation type | qualitative | 29.7% (11) | 70.3% (26) | NS |
|  | quantitative | 15.1% (11) | 84.9% (62) |  |
| clinical classes | OI type I | 7.6% (7) | 92,4% (85) | P<0.0005 |
|  | OI type III | 81.3% (13) | 18.8% (3) |  |
|  | OI type IV | 21.7% (5) | 78.3% (18) |  |

Distribution of triangular face evaluated on 131 patients

|  |  | FRONTAL BOSSING | |  |
| --- | --- | --- | --- | --- |
|  |  | yes | no | p-value |
|  | individuals | 37 | 194 | / |
| gender | male | 17.3% (18) | 82.7% (86) | NS |
|  | female | 15% (19) | 85% (108) |  |
| familiarity | yes | 15.1% (16) | 84.9% (90) | NS |
|  | no | 19.3% (11) | 80.7% (46) |  |
| mutated gene | COL1A1 | 17% (26) | 83% (127) | NS |
|  | COL1A2 | 5.3% (2) | 94.7% (36) |  |
|  | no col1 mutations | 22.5% (9) | 77.5% (31) |  |
| mutation type | qualitative | 9.1% (5) | 90.9% (50) | NS |
|  | quantitative | 16.9% (23) | 83.1% (113) |  |
| clinical classes | OI type I | 12.4% (22) | 87.6% (156) | P=0.008 |
|  | OI type III | 36% (9) | 64% (16) |  |
|  | OI type IV | 21.4% (6) | 78.6% (22) |  |

Distribution of frontal bossing evaluated on 231 patients

|  |  | DENTINOGENESIS IMPERFECTA | |  |
| --- | --- | --- | --- | --- |
|  |  | yes | no | p-value |
|  | individuals | 58 | 179 |  |
| gender | male | 21.2% (22) | 78,8% (82) | NS |
|  | female | 27.1% (36) | 72,9% (97) |  |
| familiarity | yes | 14.3% (14) | 85,7% (84) | p=0.0334 |
|  | no | 29% (20) | 71% (49) |  |
| mutated gene | COL1A1 | 19.5% (29) | 80.5% (120) | P=0.058 |
|  | COL1A2 | 33.3% (14) | 66.7% (28) |  |
|  | no col1 mutations | 33.3% (15) | 66.7% (30) |  |
| mutation type | qualitative | 35.6% (21) | 64.4% (38) | P=0.0068 |
|  | quantitative | 16.7% (22) | 83.3% (110) |  |
| clinical classes | OI type I | 16.8% (31) | 83.2% (153) | P<0.0005 |
|  | OI type III | 58.3% (14) | 41.7% (10) |  |
|  | OI type IV | 44.8% (13) | 55.2% (16) |  |

Distribution of dentinogenesis imperfecta evaluated on 237 patients

|  |  | CARDIAC ALTERATIONS | |  |
| --- | --- | --- | --- | --- |
|  |  | yes | no | p-value |
|  | individuals | 52 | 152 | / |
| gender | male | 25.3% (21) | 74.7% (62) | NS |
|  | female | 25.6% (31) | 74.4% (90) |  |
| familiarity | yes | 23.8% (20) | 76.2% (64) | NS |
|  | no | 18.8% (13) | 81.2% (56) |  |
| mutated gene | COL1A1 | 27.9% (36) | 72.1% (93) | NS |
|  | COL1A2 | 12.8% (5) | 87.2% (34) |  |
|  | no col1 mutations | 31.4% (11) | 68.6% (24) |  |
| mutation type | qualitative | 13.5% (7) | 86.5% (45) | P=0.0437 |
|  | quantitative | 29.3% (34) | 70.7% (82) |  |
| clinical classes | OI type I | 23.9% (38) | 76.1% (121) | NS |
|  | OI type III | 19% (4) | 81% (17) |  |
|  | OI type IV | 41.7% (10) | 58.3% (14) |  |

Distribution of cardiac alteration evaluated on 204 patients

|  |  | SCLERAL HUE | | |  |
| --- | --- | --- | --- | --- | --- |
|  |  | white | blue | grey | p-value |
|  | individuals | 44 | 245 | 45 | / |
| gender | male | 16.3% (24) | 68.7% (101) | 15% (22) | NS |
|  | female | 10.7% (20) | 77% (144) | 12.3% (23) |  |
| familiarity | yes | 11.2% (17) | 77% (117) | 11.8% (18) | NS |
|  | no | 10.3% (10) | 72.2% (70) | 17.5% (17) |  |
| mutated gene | COL1A1 | 7.1% (15) | 86.7% (183) | 6.2% (13) | P<0.0005 |
|  | COL1A2 | 29.9% (20) | 49.3% (33) | 20.9% (14) |  |
|  | no col1 mutations | 16.4% (9) | 52.7% (29) | 30.9% (17) |  |
| mutation type | qualitative | 27% (24) | 52.8% (47) | 20.2% (18) | P<0.0005 |
|  | quantitative | 5.8% (11) | 89.4% (169) | 4.8% (9) |  |
| clinical classes | OI type I | 11.2% (29) | 79.2% (206) | 9.6% (25) | P<0.0005 |
|  | OI type III | 22.9% (8) | 54.3% (19) | 22.9% (8) |  |
|  | OI type IV | 18.4% (7) | 50% (19) | 31.6% (12) |  |

Distribution of scleral hue evaluated on 334 patients

|  |  | SKIN ALTERATION | |  |
| --- | --- | --- | --- | --- |
|  |  | yes | no | p-value |
|  | individuals | 45 | 105 | / |
| gender | male | 25.5% (13) | 74.5% (38) | NS |
|  | female | 32.3% (32) | 67.7% (67) |  |
| familiarity | yes | 25.6% (20) | 74.4% (58) | NS |
|  | no | 33.3% (18) | 66.7% (36) |  |
| mutated gene | COL1A1 | 26.5% (27) | 73.5% (75) | NS |
|  | COL1A2 | 37% (10) | 63% (17) |  |
|  | no col1 mutations | 35% (7) | 65% (13) |  |
| mutation type | qualitative | 25.7% (9) | 74.3% (26) | NS |
|  | quantitative | 29.8% (28) | 70.2% (66) |  |
| clinical classes | OI type I | 27.7% (33) | 72.3% (86) | NS |
|  | OI type III | 37.5% (6) | 62.5% (10) |  |
|  | OI type IV | 40% (6) | 60% (9) |  |

Distribution of skin alteration evaluated on 150 patients

|  |  | JOINT HYPERLAXITY | |  |
| --- | --- | --- | --- | --- |
|  |  | yes | no | p-value |
|  | individuals | 135 | 49 | / |
| gender | male | 70.2% (59) | 29.8% (25) | NS |
|  | female | 76% (76) | 24% (24) |  |
| familiarity | yes | 70.5% (55) | 29.5% (23) | NS |
|  | no | 70% (42) | 30% (18) |  |
| mutated gene | COL1A1 | 71.4% (85) | 28.6% (34) | NS |
|  | COL1A2 | 72.7% (24) | 27.3% (9) |  |
|  | no col1 mutations | 80.6% (25) | 19.4% (6) |  |
| mutation type | qualitative | 73.7% (33) | 26.7% (12) | NS |
|  | quantitative | 71% (76) | 29% (31) |  |
| clinical classes | OI type I | 70.1% (103) | 29.9% (44) | NS |
|  | OI type III | 77.8% (14) | 22.2% (4) |  |
|  | OI type IV | 94.7% (18) | 5.3% (1) |  |

Distribution of joint hyperlaxity evaluated on 184 patients

|  |  | BMD AT LUMBAR SPINE | | |  |
| --- | --- | --- | --- | --- | --- |
|  |  | Normal | Osteopenia | Osteoporosis | p-value |
|  | individuals | 7 | 26 | 27 | / |
| mutated gene | COL1A1 | 9.8% (4) | 43.9% (18) | 46.3% (19) | NS |
|  | COL1A2 | 8.3% (1) | 41.7% (5) | 50% (6) |  |
|  | no col1 mutations | 28.6% (2) | 42.9% (3) | 28.6% (2) |  |
| mutation type | qualitative | 5% (1) | 45% (9) | 50% (10) | NS |
|  | quantitative | 12.1% (4) | 42.4% (14) | 45.5% (15) |  |
| clinical classes | OI type I | 14.6% (7) | 47.9% (23) | 37.5% (18) | NS |
|  | OI type III | / | / | 100% (1) |  |
|  | OI type IV | / | 27.3% (3) | 72.7% (8) |  |

Distribution of Bone Mineral Density evaluated on 60 children

|  |  | HEARING LOSS | |  |
| --- | --- | --- | --- | --- |
|  |  | yes | no | p-value |
|  | individuals | 80 | 186 | / |
| gender | male | 29.7% (35) | 70.3% (83) | NS |
|  | female | 30.4% (45) | 69.6% (103) |  |
| familiarity | yes | 27.9% (31) | 72.1% (80) | NS |
|  | no | 23.5% (20) | 76.5% (65) |  |
| mutated gene | COL1A1 | 30.8% (52) | 69.2% (117) | NS |
|  | COL1A2 | 26% (13) | 74% (37) |  |
|  | no col1 mutations | 31.9% (15) | 68.1% (32) |  |
| mutation type | qualitative | 28.2% (20) | 71.8% (51) | NS |
|  | quantitative | 30.4% (45) | 69.6% (103) |  |
| clinical classes | OI type I | 28% (58) | 72% (149) | NS |
|  | OI type III | 39.3% (11) | 60.7% (17) |  |
|  | OI type IV | 35.5% (11) | 64.5% (20) |  |

Distribution of hearing loss evaluated on 266 patients
