# Supplementary material for: Genotype–phenotype correlation study in 364 osteogenesis imperfecta Italian patients
Source: Eur J Hum Genet. 2019 Mar 18;27(7):1090–100. doi: 10.1038/s41431-019-0373-x (PMC6777444; doi:10.1038/s41431-019-0373-x)
Supplement: Supplementary file 3 — Additional file 3 [file 41431_2019_373_MOESM3_ESM.docx]

**Additional file 3**

Other clinical features

The presence of skin alterations (cutis laxa and/or sensitive skin) was described in the 30% of evaluated subjects (45/150; 29 with cutis laxa, 11 with sensitive skin and 5 with both clinical signs). A skin alteration was identified in 27.7% of OI type I subjects, in 40% of type IV and in 37.5% of type III patients, without any statistical significance. Moreover, no relationship emerged considering the genotype, except for a tendency of *COL1A2* quantitative mutations to be related with skin alterations (P=0.082).

Joint hyperlaxity was detected in 73.4% of patients (135/184; 70 children and 65 adults). Considering the subset of individuals with joint hypermobility, this feature was noticed in the 70.1% of OI type I evaluated patients, 94.7% of OI type IV and 77.8% of OI type III; no significant association was found. Both mutated gene and mutation type did not influence the presence of this clinical sign.

Lumbar spine bone mineral density (BMD) alterations were observed in 88.3% of children evaluated (53/60; 26 had osteopenia and 27 osteoporosis). With reference to OI type I patients with BMD alterations, 47.9% (23/48) showed osteopenia and 37.5% (18/48) osteoporosis; OI type IV was divided in 27.3% (3/11) and 72.7% (8/11); whereas about OI type III, 100% (1/1) had osteoporosis. Despite the absence of a statistical significance due to the limited number of patients, it is evident an increase of osteoporosis with a more severe OI form. No statistical significance emerged in relation to the genetic background.

Hearing impairments were recorded in 30% of the OI population (80/266; 13 children and 67 adults). This clinical sign was associated with the adult population (P<0.0005). Deafness was detected in 28% of OI type I patients, in 35.5% of type IV and in 39.3% of type III individuals; showing no significant association. No correlation with the genetic background emerged (Table 4 and additional file 3).
